# *FOXG1*-Related Syndrome: From Clinical to Molecular Genetics and Pathogenic Mechanisms

**DOI:** 10.3390/ijms20174176

**Published:** 2019-08-26

**Authors:** Lee-Chin Wong, Shekhar Singh, Hsin-Pei Wang, Chia-Jui Hsu, Su-Ching Hu, Wang-Tso Lee

**Affiliations:** 1 Department of Pediatrics, Cathay General Hospital, Taipei 106, Taiwan; 2 Graduate Institute of Clinical Medicine, National Taiwan University College of Medicine, Taipei 100, Taiwan; 3 Department of Pediatrics, National Taiwan University Hospital YunLin Branch, YunLin 640, Taiwan; 4 Department of Pediatrics, Taipei City Hospital YangMing Branch, Taipei 111, Taiwan; 5 Department of Pediatric Neurology, National Taiwan University Children’s Hospital, Taipei 100, Taiwan; 6 Department of Pediatrics, National Taiwan University College of Medicine, Taipei 100, Taiwan; 7 Graduate Institute of Brain and Mind Sciences, National Taiwan University College of Medicine, Taipei 100, Taiwan

**Keywords:** *FOXG1*, Rett syndrome, hyperkinetic movements, telencephalon, transcriptional factor

## Abstract

Individuals with mutations in forkhead box G1 (*FOXG1*) belong to a distinct clinical entity, termed “*FOXG1*-related encephalopathy”. There are two clinical phenotypes/syndromes identified in *FOXG1*-related encephalopathy, duplications and deletions/intragenic mutations. In children with deletions or intragenic mutations of *FOXG1*, the recognized clinical features include microcephaly, developmental delay, severe cognitive disabilities, early-onset dyskinesia and hyperkinetic movements, stereotypies, epilepsy, and cerebral malformation. In contrast, children with duplications of *FOXG1* are typically normocephalic and have normal brain magnetic resonance imaging. They also have different clinical characteristics in terms of epilepsy, movement disorders, and neurodevelopment compared with children with deletions or intragenic mutations. FOXG1 is a transcriptional factor. It is expressed mainly in the telencephalon and plays a pleiotropic role in the development of the brain. It is a key player in development and territorial specification of the anterior brain. In addition, it maintains the expansion of the neural proliferating pool, and also regulates the pace of neocortical neuronogenic progression. It also facilitates cortical layer and corpus callosum formation. Furthermore, it promotes dendrite elongation and maintains neural plasticity, including dendritic arborization and spine densities in mature neurons. In this review, we summarize the clinical features, molecular genetics, and possible pathogenesis of *FOXG1*-related syndrome.

## 1. Introduction

### FOXG1-Related Syndrome: A Distinct Entity from Rett Syndrome (RTT)

Rett syndrome (RTT) is a rare neurodevelopmental disorder usually affecting females, with prevalence of approximately 1 in 10,000–15,000 females. RTT is diagnosed on the basis of consensus clinical criteria [1,2]. The clinical features of typical RTT are neurodevelopmental regression at approximately 1 year of age, including acquired spoken language and hand skills, along with the development of stereotypic hand movements and gait abnormalities; for the diagnosis of typical RTT, individuals must present these four main criteria. However, those with several clinical presentations that do not fulfill all the diagnostic criteria are classified as having atypical RTT, which is subclassified into three categories according to each distinct clinical entity. These categories include congenital variant, early seizure variant, and preserved speech variant [1]. Most individuals (80–90%) with typical RTT are associated with methyl-CpG-binding protein 2 (*MECP2*) mutations. In addition to *MECP2* mutations, other genetic mutations, such as forkhead box G1 (*FOXG1*) and cyclin-dependent kinase-like 5 (*CDKL5*), have been identified in atypical RTT variants [1]. In particular, patients with *FOXG1* mutations are associated with congenital RTT, wherein these patients have disease onset and global development delay from early infancy, i.e., before reaching 6 months of age [3].

*FOXG1*, located on chromosome 14q12 (chr14: 28,765,388-28,770,27; GRCh38/hg38) and 4890 bp in size, has one coding exon and belongs to the forkhead (FOX) family of genes. It consists of a 489-amino-acid protein in humans and contains a highly conserved domain spanning from the forkhead binding domain (FBD) to the C-terminus and variable N-terminus [2]. Furthermore, *FOXG1* encodes a transcription factor, playing an essential role in the ventral telencephalon development. *FOXG1* alterations include duplications, deletions, frameshifts, and point mutations [4,5,6,7]. The first case of *FOXG1* mutation was reported in 2005 when a girl presented with severe cognitive impairment, the agenesis of corpus callosum, and microcephaly [8]. Although patients with *FOXG1* alterations were initially described to have congenital RTT variants [1,3], there have been increasing reports on individuals harboring *FOXG1* mutations. This allows further expansion and delineation of the clinical phenotypes of *FOXG1* mutations, thus progressing parting from RTT. Compared to RTT, individuals with *FOXG1* mutations generally lack ‘eye gazing/pointing’, are more severe in language, ambulation, social interaction, and sleeping disturbance, and most importantly, lack of the regression as observed in RTT [6,9,10,11,12,13].

It is now considered that individuals harboring mutations in *FOXG1* belong to a distinct clinical entity, which is termed “*FOXG1*-related encephalopathy”. There are two clinical phenotypes/syndromes identified in *FOXG1*-related encephalopathy, duplications and deletions/intragenic loss-of-function mutations. In children with *FOXG1* deletions or intragenic mutations, the recognized clinical core features include microcephaly, developmental delays, severe cognitive disabilities, absence or minimal language development, early-onset dyskinesia and hyperkinetic movements, stereotypies, epilepsy, and cerebral malformation [14,15]. In contrast, children with *FOXG1* duplications are typically normocephalic and have normal brain or nonspecific changes of brain, as detected on magnetic resonance imaging (MRI). Additionally, they have different clinical characteristics in terms of epilepsy, movement disorders, and neurodevelopment compared with children with deletions or intragenic mutations of *FOXG1*.

To date, there are more than 120 different mutations, including deletions, intragenic mutations, and duplications, of *FOXG1* have been reported worldwide [4,6,10,11,12,16,17,18,19,20,21,22,23,24,25,26,27]. In addition to clinical studies, both in vivo and in vitro studies have been performed to delineate the function of *FOXG1* and pathogenic mechanisms underlying this syndrome. FOXG1 is expressed mainly restricted in the telencephalon. It plays a determining role in the development and regionalization of the anterior brain [28], as well as cortical lamination and the forming of the corpus callosum [29]. It is involved in promoting neural precursor proliferation and regulates their differentiation [30,31]. It is critical in the balance of inhibitory/excitatory neurons and their markers [32,33]. Furthermore, it also promotes survival and neurite elongation in post mitotic neuron, maintaining the neural plasticity, including dendritic arborization and spine densities in mature neurons [34,35,36,37], which are important for high-grade function.

In this review, we have briefly summarized the clinical features, molecular genetics, and pathogenesis of *FOXG1*-related syndrome.

## 2. Clinical Features of *FOXG1*-Related Syndrome

Heterozygous pathogenic variants in *FOXG1* cause a severe neurodevelopmental encephalopathy characterized by clinical features, such as severe global delay, cognitive impairment, movement disorders, autistic behavior (especially poor eye contact), and epilepsy. There are two clinical syndromes identified in *FOXG1* mutations: (1) deletions/intragenic mutations and (2) duplications. The clinical characteristics of *FOXG1* duplications and deletion or intragenic mutations are summarized in Table 1.

### 2.1. FOXG1-Related Syndrome: Deletions/Intragenic Mutations

Individuals with *FOXG1* deletions or intragenic loss-of-function mutations typically present with normal or borderline normal head circumference at birth, followed by the development of severe postnatal microcephaly. Starting from early infancy, the individuals develop global development delay, hypotonia, and cognitive impairment, which are mostly accompanied by irritability, poor feeding, and visual impairment. Besides, the individuals typically have minimal or absence of speech and do not attain ambulation.

#### 2.1.1. Movement Disorders

Early-onset hyperkinetic movement disorder is the cardinal feature of this syndrome. It is characterized by choreoathetosis, dystonia and orolingual/facial dyskinesias, and myoclonic jerk, which commonly develop by 1 year of age [9,11,38]. Stereotypies are present in 50–87% of the individuals [6,9,10]. The hand stereotypies often involve objects, and hand-mouthing is most commonly described in these individuals [10]. In comparison with typical RTT, in which the stereotypies are ubiquitous among the patients and midline hand wringing is the most common one [39], nearly 50% of individuals with *FOXG1* deletions demonstrate stereotypies and more commonly involve objects with hands, including mouthing of toys, grasping clothes or objects, and nail biting, whereas midline hand wringing is rarely observed [9]. The movement disorders are present while at rest but may worsen while attempting voluntary movements [10]. They generally cause functional impairment, leading to disturbance in playing, eating, voluntary movement, and even sleeping. The progression of movement disorders is variable; although some remain relatively stable, some may worsen over time, or evolve from hyperkinetic to predominantly hypokinetic movements [13]. However, there is no status dystonicus observed in these individuals, as opposed to neurodegenerative diseases or genetic hyperkinetic disorders [40]. Generally, symptomatic medications that target movement disorders are not beneficial, although some individuals have been reported to be responsive to levodopa and tetrabenazine [9].

#### 2.1.2. Epilepsy

Approximately 68–87% of individuals with *FOXG1* deletions have epilepsy [6,12]. The onset of seizures typically occurs in early childhood, commonly before 2–3 years of age, and it ranges from 2 days to 14 years; however, the age of onset of seizures is generally later compared with that in individuals with *FOXG1* duplications, which mostly occur at 3–7 months of age [6,10,12]. Seltzer et al. [12] showed that the mean age of diagnosis of epilepsy in *FOXG1* duplications is significantly younger than that in deletion/intragenic mutations (7.4 months of age vs 22.3 months of age). The variable seizure types include infantile spasms, generalized tonic-clonic seizures, myoclonic seizures, and focal seizures. They are often refractory to medications, but status epilepticus is rare. Meanwhile, infantile spasms are less commonly seen in these individuals and are typically refractory compared with those in individuals with *FOXG1* duplication, wherein the spasms are often responsive to adrenocorticotropic hormone (ACTH) therapy. The spasms in these patients may later evolve into Lennox–Gastaut syndrome, a severe form of epilepsy [6]. In a recent cohort study involving 45 individuals with *FOXG1* deletion/intragenic mutations, 77.8% (35/45) had seizures, with generalized tonic-clonic seizures being the most common type. Among the 35 individuals with epilepsy, only 17 (48.6%) had refractory seizures [10]. Moreover, the electroencephalogram (EEG) may show focal or multifocal epileptiform discharges without a specific pattern. In general, there is no particular epilepsy syndrome in individuals with these mutations. Moreover, there is no obvious correlation between genotypes and seizure types [12,15,41].

#### 2.1.3. Brain Images 

Individuals with *FOXG1* deletions or intragenic mutations have a spectrum of structural brain anomalies, ranging from hypogenesis of the corpus callosum to frontal pachygyria combined with delayed myelination in its most severe form [10,14].

In a cohort study consisting of 37 individuals with *FOXG1* heterozygous mutations by Vegas et al. [10], there were three patterns of gyration identified: (1) Mildly simplified or normal gyration, (2) moderately simplified gyration, and (3) frontal pachygyria. The structural abnormalities of the corpus callosum is another hallmark of brain MRI findings in these children, but they are variable, ranging from hypoplasia (mostly in the genu part) to the agenesis of the corpus callosum. Delayed myelination is also common, but this typically improves over time. Intriguingly, serial MRI studies in one child showed the evolution of gyration pattern with age—frontal pachygyria in infancy evolved to mild gyral simplification in early childhood. This finding suggests that these two cortical patterns reflect different maturation of subcortical white matter over time, instead of two different morphologies. Furthermore, for different genotypes, patients harboring frameshift and nonsense mutations in the N-terminus of *FOXG1* show the most severe MRI anomalies.

#### 2.1.4. Other Comorbidities

Apart from cognitive impairment, impairment of the visual system has been described in these patients. The ocular impairment includes strabismus, which is most commonly described, oculo-mandibular synkinesias, and small optic disks [6,27]. Furthermore, they may also have high-level visual dysfunctions resembling ‘‘blindsight” [27].

Sleep disturbance is common in these children, and 64.3–72.7% of the children have sleep problems [10,15], including difficulties in falling asleep with irritability and inconsolable crying, inappropriate laughing, or frequent nocturnal awakenings. Nonetheless, the sleep problems may improve with age.

Gastrointestinal issues such as gastroesophageal reflux and constipation are also common and may be severe, thus requiring a feeding tube or even surgical intervention. Additionally, breathing abnormalities are variable [15].

#### 2.1.5. Genetic Mutations and Genotype–Phenotype Correlation

**Figure 1:**
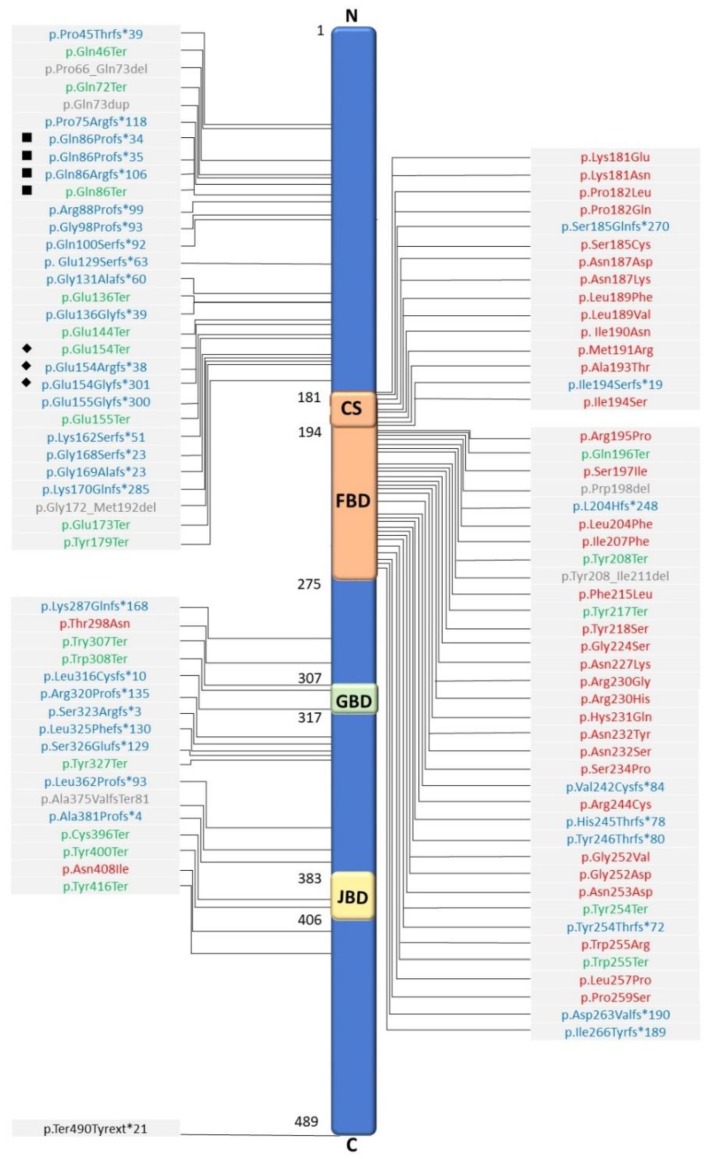
FOXG1 protein domains and distribution of variants. Published *FOXG1* variants in a schematic illustration; the N-terminal domain, forkhead binding domain (FBD, amino acids 181-275), forkhead domain conserved site 1 (cs, amino acids 181-194), Groucho-binding domain (GTBD, amino acids 307–406), JARID1B-binding domain (JBD, amino acids 383–406), and C-terminal domain of FOXG1 protein are indicated. The published mutation variants include missense variants (red), frameshift (blue), nonsense (green), in-frame (gray), and stop loss (black) variants. The mutations are distributed in all protein domains. Most missense variants (red) cluster in the FBD, including cs, and are not found in the N-terminal domain. Frameshift variants (blue) are predominantly located in the N-terminal, while nonsense variants are found in all domains except the cs domain. Variants of two hot spot mutations are shown: ■ are variants of mutations at c.256, and ◆ are variants of mutations at c.460.

Since the first individual with *FOXG1* mutation described in 2005 [8], there have been up to 100 variants of *FOXG1* identified [3,6,7,9,10,11,12,13,14,15,16,24,42,43,44,45,46,47,48,49,50,51,52,53,54,55,56,57,58,59,60] (Table S1). The mutations include large, frameshift, in-frame, missense, and nonsense mutations (Figure 1). Typically, most mutations are de novo, although cases of the mutations inherited from clinically unaffected parents with somatic mosaicism have been reported [9,59]. The mutations are distributed all along the *FOXG1* gene and all protein domains (Figure 1). Two hot spots for mutations are c.256dupC and c.460dupG, which are located in the N-domain, and each locus has seven repeated sequences of cytosines (CCCCCCC) and guanines (GGGGGGG), respectively, that may be prone to replication errors [6,10,12,15]. Importantly, there are also cases harboring deletions, located not directly in *FOXG1* but in a downstream region, that include elements involved in regulating *FOXG1* transcription. These mutations cause misregulation of the *FOXG1* and result in *FOXG1* haploinsufficiency [11,61,62]. As increasing variants have been discovered and the phenotypes of *FOXG1* variants have been expanding in recent years, efforts have been made to delineate the genotype–phenotype correlation. These studies share some similarities, but also have conflicting results. Overall, the mutations in the N-terminal are more likely to be associated with severe phenotypes, and mutations in the C-terminal are associated with milder phenotypes.

A cohort study performed by Mitter et al. [6] involved 83 individuals; those with the more severe phenotypes were found to harbor truncating mutations in the forkhead domains (except conserved site 1) and the N-terminal, whereas those with milder phenotypes were found to harbor missense mutations in forkhead conserved site 1. Moreover, the most significant differences were related to motor and speech developments, whereas there were only borderline differences found in terms of brain structural anomalies. Another cohort study involving 45 individuals focused on brain MRI findings; it was found that individuals with N-terminal mutations and large deletion had the most severe clinical features and MRI abnormalities, whereas those with forkhead binding domain (FBD) or C-terminal mutations had milder phenotypes. However, of note, the two hot spot mutations, c.256dupC and c.460dupG, may have highly variable features that include a variable degree of corpus callosum anomalies and epilepsy severities. Thus, this suggests that factors other than primary genetic mutation (e.g., environment factors and epigenetic) may play crucial roles in a clinical phenotype [10].

### 2.2. FOXG1-Related Syndrome: Duplication

There are less reports on *FOXG1* duplications [4,12,17,18,19,20,21,23,25,63]. Nevertheless, individuals with *FOXG1* duplication have much distinct features, such as epilepsy, movement disorders, head circumference, and brain MRI abnormality, distinguishable from those of *FOXG1* deletions/intragenic mutations. These individuals typically have normal head circumferences. Although they have global developmental delay, they are not always as seriously impaired as those with *FOXG1* deletions/intragenic mutations. Accordingly, individuals with *FOXG1* duplications often have absent/delayed language development and autistic behaviors. In spite of that, fine motor skills are not as severely affected as in the case of *FOXG1* deletions/intragenic mutations. Additionally, they are more likely to attain ambulation and may be able to walk within the first two years compared with individuals with *FOXG1* deletions/intragenic mutations.

#### 2.2.1. Movement Disorders

Although these individuals typically do not demonstrate hyperkinetic movements, which are the peculiar features of those with *FOXG1* deletions/intragenic mutations [4], there are rare case reports describing stereotypies and dyskinesia in such patients [38].

#### 2.2.2. Epilepsy

Individuals with *FOXG1* duplications also have epilepsy, which is much different from that in *FOXG1* deletions/intragenic mutations. The age of onset of epilepsy is usually younger, and it is in early infancy, mostly 3–7 months of age. Further, the individuals more specifically present with infantile spasms, although other seizure types, such as focal seizures, myoclonic seizures, and generalized tonic-clonic seizures, have also been observed. EEG frequently shows hypsarrhythmia. In contrast to those with *FOXG1* deletions/intragenic mutations, infantile spasms are typically responsive to ACTH therapy. Moreover, the spasms do not evolve into other epilepsy syndromes nor develop into other seizure types [6,12]. The subsequent follow-up EEG ranges from normal to focal or multifocal epileptiform discharges.

#### 2.2.3. Brain Images

The brain MRI in individuals with *FOXG1* duplications is typically normal; however, nonspecific findings, such as mild brain atrophy, thin corpus callosum, and delayed myelination, have been reported [18,25].

#### 2.2.4. Genetic Mutations

To date, up to 21 cases of *FOXG1* duplications have been reported in the literature (Table 2). The mutations were typically de novo and were mostly identified via comparative genomic hybridization array. The duplications ranged 3.1–33.9 Mb on 14q, encompassing the *FOXG1* (Table 2) [4,12,17,18,19,20,21,23,25,63,64]. However, genotype–phenotype correlation cannot be well described due to limited case numbers.

## 3. In Vitro Study of Possible Molecular Functions of FOXG1 in Neurons and Other Tissues

FOXG1 is a transcription factor that interacts with multiple signaling pathways and is essential for the proliferation of the progenitor cells of the cerebral cortex. FOXG1 interacts and cooperates with various proteins to express and repress different genes. It also inhibits the expression of a gene by attaching to specific proteins crucial for the gene expression, or by directly binding to the promoter region of the gene. Changes in the expression pattern of FOXG1 can alter the mode of mechanisms, leading to abnormal phenotypes.

**Figure 2:**
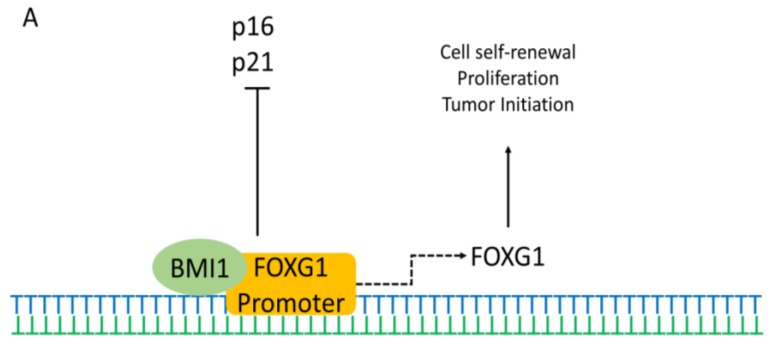

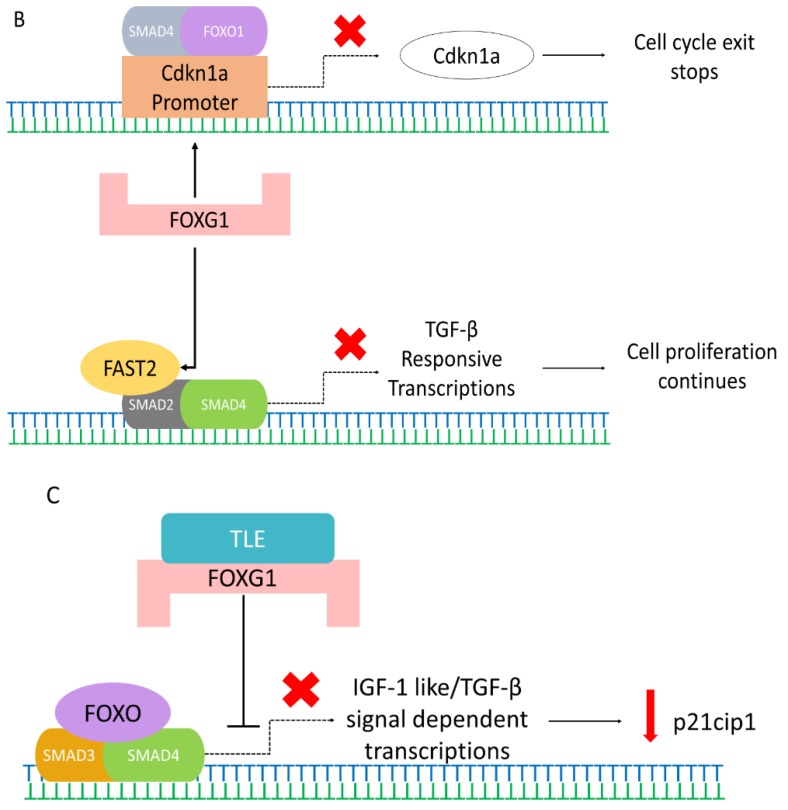
Possible pathways of FOXG1 controlling the different mechanisms in the cell. (**A**) BMI1 (green) interacts and cooperates with FOXG1 promoter (orange), which initiates FOXG1 expression. This interaction also controls cell self-renewal, proliferation, differentiation, and tumor growth. Simultaneously, it also inhibits cell cycle inhibitors p21 and p16. (**B**) FOXG1 (pink) inhibits the expression of Cdkn1a by associating and attaching to the SMAD4 (grey) and FOXO1 (purple) complexes at Cdkn1a promoter to prevent the cell cycle exit and stop differentiation. Also, FOXG1 interacts with FAST2 to inhibit the FAST2 (yellow)-SMAD2 (dark grey)-SMAD4 (green) complex formation, thereby repressing the TGF-β responsive transcriptions and allowing cell proliferation. Cross (red) indicate the transcriptional loss. (**C**) TLE-FOXG1 complex represses the FOXO (purple)-SMAD3 (orange)-SMAD4 (green)-mediated transcription of p21cip1 initiated by IGF-1like/TGF-β signal, which inhibits apoptosis while promotes growth and proliferation.

In vitro studies have shown that FOXG1 is expressed in forebrain neural stem cells (NSCs) throughout life. The interaction of FOXG1 with different proteins shows its dependence on different proteins for regulating various mechanisms. Previous studies have demonstrated that the polycomb factor Bmi-1 is necessary for NSC self-renewal and that it also represses the cell cycle inhibitors p16 and p21. Recent studies have shown that Bmi-1 and FOXG1 can cooperate to maintain the NSC multipotency and self-renewal. Bmi-1 interacts with *FOXG1* promoter and initiates FOXG1 expression (Figure 2A) [65,66]. The interaction can also inhibit p16 and p21, leading to tumor formation.

FOXG1 also interacts with FAST2 and inhibits the expression of transforming growth factor-beta (TGF-β). FOXG1 interacts with FAST2 to inhibit the FAST2-SMAD2-SMAD4 complex formation, thereby repressing TGF-β-responsive transcriptions and allowing cell proliferation. (Figure 2B) [67]. FOXG1 may also repress the expression of another TGF-β-induced protein (p15) and upregulate the expression of a TGF-β-repressed protein (cyclin A). Therefore, FOXG1 is a general TGF-β signaling inhibitor and may play an essential part during brain development [68]. Furthermore, overexpression of FOXG1 may inhibit gliogenesis, promote neurogenesis, and may significantly stimulate outgrowth of neurite [69]. The overexpression of FOXG1 may also result in enduring NSC compartment enlargement via increased stem cell self-renewal and promoting progenitor survival [69]. Furthermore, the Shh signaling pathway also plays an important role in the development of the telencephalon, and previous studies have shown the interaction of FOXG1 with the Shh signaling pathway [70].

In addition to the involvement in neurodevelopmental disorders, FOXG1 is also shown to be associated with tumor formation. The overexpression or loss-of-function of FOXG1 can lead to both malignant transformation and tumorigenesis. 

**Figure 3:**
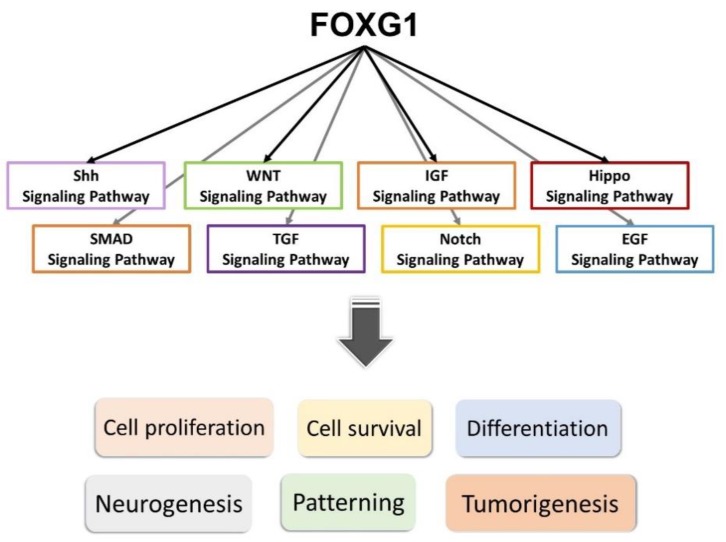
FOXG1 interacts with members of different signaling pathways—including the Shh, WNT, IGF, Hippo, SMAD, TGF, Notch, and EGF signaling pathways—that are important in neurodevelopment, and promotes proliferation, survival, and differentiation of neurons or other cell types. It exerts critical impacts on neurogenesis and patterning of the telencephalon, and may also be associated with tumorigenesis.

Previous studies have shown the potential role for FOXG1 expression and activated Notch signaling in impeding proneural differentiation in tumors, and also the interaction of the FOXG1/Notch pathway during neurogenesis [71]. More recent studies have shown that decreased expression of FOXG1 might result in the downregulation of most Notch pathway genes (Figure 3) [71]. A recent study has also shown that knockdown of *FOXG1* in brain tumor-initiating cells might result in downregulation of neural progenitor and proliferation markers and upregulation of astroglial differentiation genes [72]. A study in conditional knockout of *Foxg1* in mouse hair cells has also shown the interaction of Foxg1 with the IGF1, Wnt, and Hippo pathways, etc. [73]. These indicate that Foxg1 may function via a complex interaction with several members of different pathways in neurodevelopment, including the Wnt, Notch, SMAD, IGF, and Shh signaling pathways (Figure 3).

Previous studies also showed that the expression of FOXG1 was high in embryonal and small cell components in some tumors [74,75], and was correlated with gliomagenesis and its malignancy [76]. FOXG1 repressed the expression of cyclin-dependent kinase inhibitor p21cip1 by inhibiting the FOXO/SMAD3/4 complex transcriptional activity (Figure 2C) [75]. FOXG1 overexpression also considerably reduced cleaved-caspase-9/8/3 and caspase-9/8/3 expression [76]. Consequently, the expression of the caspase family was elevated in loss-of-function of *FOXG1* mutations, initiating cell apoptosis. This indicates that FOXG1 expression may have a direct regulation on the caspase family and may act as a negative regulator of apoptosis of tumor cells, inhibiting cell apoptosis.

In contrast, FOXG1 expression is low with an increased expression of the oncogene nuclear receptor coactivator amplified in breast cancer 1 (AIB1) in breast cancer cells [77]. In these cells, FOXG1 may function as a proapoptotic factor by reducing the expression of AIB1 oncogene through suppression of AIB1 coactivator transcription complex formation. Therefore, depending on the stimulus from the different cellular environments, FOXG1 may act as a pro- or antiapoptotic factor. FOXG1 may act through different pathways by which to achieve cell proliferation, survival, differentiation, or apoptosis.

## 4. *FOXG1*-Related In Vivo Models and Possible Pathogenic Mechanisms

*FOXG1* is evolutionarily conserved, and is required in vertebrate neurodevelopment [2]. The amino acid sequences from FBD to the C-terminal domain are highly conserved (96%) among species [78], whereas the N-terminal domain is quite variable. The expression of FOXG1 is not ubiquitous. It is expressed in the forebrain at all developmental stages, as well as adulthood. In the developing brain of a human embryo, the earliest expression of FOXG1 is at early (postconception 3 weeks + 4 day) telencephalic neuroepithelial progenitors. At the mid corticogenesis stage (postconception 7 weeks + 2 days), FOXG1 is strongly expressed in the ventricular zone, subventricular zone, and cortical plate [79]. In mice, the pattern of expression of Foxg1 is similar to humans. It first appears in the anterior edge of the neural plate within ectoderm around the five-somite stage. The expression gradually expands caudally occupying a significant proportion of the forebrain. It is then highly expressed in the telencephalon and retina [2,80,81]. In zebrafish, it is first expressed around 12 h, at the margin of the telencephalon, and then expanded at 14–18 h [82]. In addition, Foxg1 is also expressed in other neural sensory tissues such as the retina, optic chiasm, ear, and olfactory placode [2,83,84].

### 4.1. The Functions of FOXG1 in Neurodevelopment

**Figure 4:**
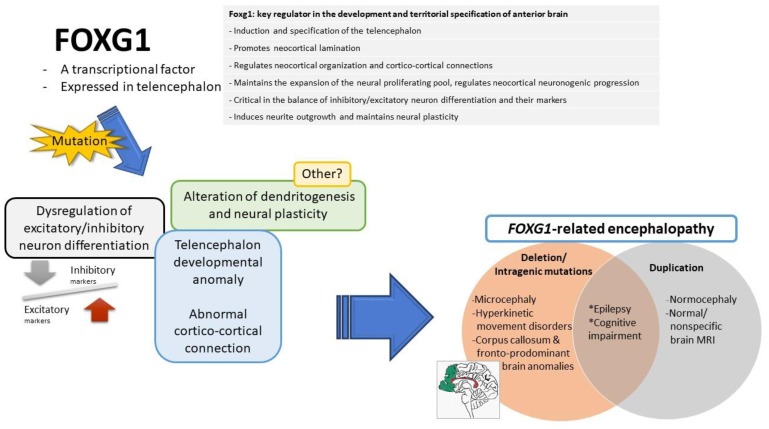
The possible functions of FOXG1 (right upper), which have been demonstrated in in vivo animal models: The mutations of *FOXG1* gene may lead to possible pathological events (left), including dysregulation of excitatory/inhibitory neuron differentiation, telencephalon developmental anomaly, abnormal cortico-cortical connection, and alteration in dendritogenesis and neural plasticity. These may be the underlying possible pathomechanisms leading to *FOXG1*-related encephalopathy (right lower).

FOXG1 plays a pleiotropic role in the development of the brain (Figure 4) and is a key regulator in the development and territorial specification of the anterior brain [28,31]. It is involved in the induction of the telencephalon and specification of the telencephalic field, establishing the spatial subdivisions within the telencephalic dorsoventral and mediolateral compartments [2]. It regulates the production of ventralizing signals and promotes telencephalic precursors sensitive to ventralizing signals [28,70]. It promotes neocortical lamination, switching cortical progenitor cells from the early generation of the primordial plexiform layer to the late production of the cortical plate, and contributes to cortical area profile shaping [85,86].

In addition, it maintains the expansion of the neural proliferating pool [87], properly tunes the neuronal differentiation rate, and regulates the pace of neocortical neurogenic progression. Constitutive suppression of FOXG1 in cortical progenitor cells leads to their differentiation as Cajal–Retzius cells, the earliest-born neuron in the developing cerebral cortex [86]. FOXG1 is dynamically expressed in later-born neurons (transient downregulation and later re-expression), to coordinate their progression through the multipolar pyramidal precursor, and subsequent migration toward the cortical plate [88], as well as the proper timing of upper-layer and lower-layer neurogenesis [89]. In post-migratory neurons, it remains highly-expressed and is required for the formation of the corpus callosum and cortical laminar structure [29]. It is also critical in the balance of inhibitory/excitatory neurons and their markers, as is shown in human cerebral organoids [32,33].

Beyond its complex impacts in cortical arealization, lamination, and neurogenesis, FOXG1 is crucial in dendritogenesis. It induces neurite outgrowth [69], promotes dendrite elongation, and maintains neural plasticity, including dendritic arborization and spine densities in a mature neurons [34,36].

Furthermore, Foxg1 promotes optic fissure closure, regulates retinal axon pathfinding, and facilitates the crossing of retinal ganglion cell axon during development [83,84,90]. It is also important for the proper formation of the inner ear and the olfactory system [91,92].

### 4.2. The Possible Pathogenic Mechanisms of FOXG1-Related Syndrome

The functions of Foxg1 have been progressively delineated via in vivo and in vitro studies. There are some manifestations in animal models of *Foxg1* mutations that resemble human phenotypes, which could partially explain the underlying pathogenic mechanism of *FOXG1*-related syndrome. However, there remains much to explore on the pathogenesis leading to *FOXG1*-related syndrome.

Herein, we summarize the important findings regarding abnormal phenotypes caused by alteration of *Foxg1* in in vivo studies that could explain the possible pathogenic mechanisms underlying *FOXG1*-related syndrome.

#### 4.2.1. Reduced Volume of Hemisphere with Disrupted Brain Morphology

The role of Foxg1 as a key regulator in development and specification has been well demonstrated in animal studies. A knockout mice research performed by Xuan et al. first showed the fundamental role of Foxg1 in brain development [93]. There was a dramatically decreased volume of cerebral hemispheres and lack of ventral telencephalic structures in *Foxg1* knockout mice. In morpholino knockdown of *Foxg1* in embryos of zebrafish, there was an absence of subpallial (ventral) cells, which was caused by both their transformation into pallial progenitors and partial displacement into the hypothalamic territory [70], indicating the critical role of Foxg1 in telencephalon development and brain territorial specification. Furthermore, during cortical lamination, *Foxg1* was initially suppressed and later reactivated in order for the neuron to migrate to cortical plates. When multipolar cells failed to reexpress Foxg1, they permanently lost their ability to enter into the cortical plate [88]. These findings may link to brain structural defects in patients with *FOXG1* deletion/intragenic mutations. In these patients, they have significant microcephalies and hypoplastic forebrain, ranging from mildly simplified or normal gyration to frontal lobe predominant-pachygyria [10]. Intriguingly, even though switching off of *Foxg1* in pioneer neurons is mandatory for their differentiation as Cajal–Retzius cells, and the suppression of *Foxg1* is required during the early process of neuronal migration [88], patients with *FOXG1* duplication are typically normocephalic with normal brain or nonspecific brain structural anomalies. There are probably other possible mechanisms leading to brain development, particularly neuron migration in the context of *FOXG1* duplication.

Further, in mouse models, Foxg1 has been shown to be highly expressed in postmigratory projector neurons. It is essential for cortical layer and corpus callosum formation. There were significantly thinner cortices, enlarged ventricles, and absent corpus callosum in conditional knockout mice [29]. This finding could correlate well with the brain structural changes in *FOXG1* deletion/intragenic mutations, in which corpus callosum agenesis/dysgenesis is one of the hallmarks [10].

In addition, hippocampus atrophy had also been reported in *FOXG1*-related encephalopathy [37,51]. Hippocampal structural anomaly has been demonstrated in studies on the hippocampal dentate gyrus (DG) on conditional knockout of *Foxg1* in mice [29]. There were subgranular zone losses and a severely damaged secondary radial glial scaffolds, leading to primordial granule cells’ impaired migration. Furthermore, formation of the suprapyramidal and infrapyramidal blades of the DG was severely disrupted [37].

#### 4.2.2. Imbalance of Inhibitory/Excitatory Neurons and Their Markers

A proper level of FOXG1 has been found to be crucial for the balance of inhibitory/excitatory neurons and their markers during neuronal differentiation [32,33]. Patriarchi et al. showed that in induced pluripotent stem cell (iPSC)-derived neurons generated from both *FOXG1*+/− patients and *Foxg1*+/− mouse brain, there were increased inhibitory synaptic markers and decreased levels of excitatory synaptic markers [33]; furthermore, there was also increased levels of GluD1 (orphan glutamate receptor δ-1 subunit). GluD1 is a synaptic cell adhesion protein that regulates synaptic differentiation. It shifts the balance between excitatory and inhibitory synapses toward the latter [94]. Interestingly, there was also an increased level of GluD1 in iPSC-derived neurons obtained from patients with mutations in both *MECP2* and *CDKL5* [95]. This shared pro-inhibitory imbalance during neuronal differentiation might be implicated in the pathogenesis of clinical overlap among *FOXG1*-, *CDKL5*- and *MECP2*-related syndromes.

Moreover, in a study using a mouse model that focused on visual function [27], the *Foxg*^1+/Cre^ mice had a significant reduction in response amplitude and visual acuity of visually-evoked potentials compared with wild-type littermates. The morphological investigation showed abnormalities in the organization of excitatory/inhibitory circuits in the visual cortex with no alterations in the retinal structure. This finding could well correlate to the high-level visual dysfunctions in individuals with *FOXG1* intragenic mutations.

However, there remains much to be clarified. On top of all is that as the overexcitation may contribute to the pathogenesis of epilepsy in children, increased inhibition of neurons in such situations seems to be conflicting with the early onset of epilepsy in *FOXG1*-related syndrome patients.

#### 4.2.3. Alteration in Dendritogenesis and Neural Plasticity

*Foxg1* also exerts a critical impact on dendritogenesis and maintains the neural plasticity [34]. Dendrite dysmorphogenesis has been reported to be associated with several neurological and neurodevelopment disorders, such as autism, Alzheimer’s disease, schizophrenia, anxiety, and depression [96], as well as RTT with *MECP2* mutations [97].

In vitro and animal studies have shown that the overexpression of *Foxg1* leads to dendrite elongation, whereas the knockdown or knockout of *Foxg1* leads to reduced axon and dendrite length, as well as dendrite branching and spine densities [34,36]. The different dendritic morphologies caused by alteration of *Foxg1* dosage could probably contribute to distinctive clinical characteristics between *FOXG1* duplication and deletion/intragenic mutation. In particular, in West syndrome patients with *FOXG1* duplication, hypsarrhythmia in EEG might be related to the eccentric enlargement of the dendritic trees. Furthermore, the study on the conditional knockout of *Foxg1* in mice showed defects in social and cognitive behaviors, which could be correlated to the clinical symptoms of patients with *FOXG1*-related encephalopathy [34]. However, because these studies were only performed in in vitro and animal models, the role of *FOXG1* in dendritogenesis of human remains to be explored and delineated.

## 5. Conclusions

As shown in in vitro and animal studies, Foxg1 is an essential transcription factor in neurodevelopment. It regulates different cellular and molecular mechanisms, which are crucial for cell and neuronal survival. A fine balance of Foxg1 is necessary for the proliferation, specification, and pattern formation of the nervous system. Therefore, changes in the expression pattern of Foxg1 may lead to numerous cellular abnormalities. These may link to underlying pathogenesis of *FOXG1*-related encephalopathy in humans. However, there is still much to be explored to further delineate the pathogenesis of *FOXG1*-related encephalopathy.

## Figures and Tables

**Table 1:** The clinical characteristics of patients with *FOXG1* deletion/intragenic mutations and *FOXG1* duplications.

Clinical Features	Deletion/Intragenic Mutations of *FOXG1*	Duplication of *FOXG1*
Neurodevelopment	Severe global delay	Global delay, but variable severities
Speech	Absence or minimal	Delay, but may produce beyond words
Ambulation	Typically not acquired	Impaired, but may have ability to walk
Social contact	Impaired	Impaired
Sleep disorder	Present	Present
Visual impairment	High-level visual dysfunctions, strabismus, small optic disc, etc.	+/−
Breathing abnormalities	+/−	+/−
Movement disorder: Dyskinesia, hyperkinetic movements	Starts from early childhood	+/−
Stereotypies	Present	+/−
Microcephaly	Typically normal or borderline small at birth, evolving to severe microcephaly in infancy	+/−
Brain MRI	Corpus callosum hypogenesis/agenesis; forebrain anomaly; delayed myelination	Typically normal
Epilepsy	Onset in early childhood, variable seizure types, often refractory to treatment	Infantile spasms, mostly responsive to adrenocorticotropic hormone (ACTH)

**Table 2:** The published cases of 14q duplication encompassing *FOXG1*.

Reference	Sex	Genomic Locus/Coordinates (Genomic Mapping Reference)	Size (Mb)	Inheritance
[17]	F	Chr 14: 25.97–30.42 Mb (hg18)	4.45	De novo
[18]	M	Chr 14: 26908812–30254928 (hg18)	3.3–3.4	De novo
	F	Chr 14: 19582682–29076500 (hg18)	9.4–11	De novo
	M	Chr 3: 191656626–199287624 Chr 14: 19582682–33275612 (hg18)	7.6–8.4 13.7–18.4	Mother: Chr 3; 14 translocation
	M	Chr 14: 28217364–34635622 (hg18)	6.4	De novo
	M	Chr 14: 27474978–30603041 (hg18)	3.1	De novo
	M	Chr 14: 19508845–34063670 (hg18)	14.5	De novo
	M	Chr 14: 28257153–35048345 (hg18)	6.8	N/A
[19]	F	Chr 14: 27165797–30192375 (hg18)	3.3	De novo
[20]	M	Chr14: 19.365Kb–30.359Kb (hg18)	11	Small extranumerary marker (with part of duplication) from maternal balanced translocation involving chr 14 and 15.
	M	Chr 14: 27409kb-30603kb (hg18)	3.2	De novo
[21]	F	Chr14: 19761035–30941609, Mosaic duplication (N/A)	11.1	Maternal uniparental disomy 14 and mosaic small marker of paternal origin containing the proximal long arm of chr 14.
[12]	M	Chr 14: 26169335–30237575 (hg18)	4.1	De novo
	M	Chr 14: 19528022–35193884 (hg18)	15.6	De novo
	M	Chr 14: 21927637–55833232 (hg18)	33.9	De novo
	M	Chr 14: 20555119–33885364 (hg18)	13.3	De novo
	F	Chr 14: 26558014–30980441 (hg19)	4.4	De novo
[23]	F	Chr 14: 19365051–31212370 (hg18)	11.84	De novo
[4]	M	Chr14: 19043189–33814746 (N/A)	14.8	De novo
[63]	F	Chr14: 20516277–38826881 (hg17)	18.3	De novo
[25]	F	Chr14: 20203610–40396835 (hg19)	20	De novo

## Supplementary Materials

Supplementary materials can be found at https://www.mdpi.com/1422-0067/20/17/4176/s1.

## Abbreviations

ACTH	adrenocorticotrophic hormone
AIB1	amplified in breast cancer 1
BMI1	B-cell specific moloneymurine leukemiavirus insertion site 1
bp	base pair
CDKL5	cyclin-dependent kinase-like 5
Cdkn1a	cyclin dependent kinase Inhibitor 1A
cs	Conserved site
DG	dentate gyrus
EEG	electroencephalogram
EGF	epidermal growth factor
FBD	forkhead binding domain
FOXG1	forkhead box G1
FOXO	Forkhead box protein O
GluD1	glutamate receptor δ-1 subunit
GTBD	Groucho-binding domain
IGF-1	Insulin-like growth factor 1
iPSC	induced pluripotent stem cells
JBD	JARID1B-binding domain
Mb	Megabyte
MECP2	methyl-CpG-binding protein 2
MRI	magnetic resonance imaging
N/A	Not available
NSCs	neural stem cells
p21cip1	p21 cyclin-dependent kinase inhibitor
RTT	Rett syndrome
TGF-β	transforming growth factor-beta
TLE	transducin-like enhancer of split
Wnt	Wingless/Integrated
