# Supplementary material for: FOXG1-Related Syndrome: From Clinical to Molecular Genetics and Pathogenic Mechanisms
Source: Int J Mol Sci. 2019 Aug 26;20(17):4176. doi: 10.3390/ijms20174176 (PMC6747066; doi:10.3390/ijms20174176)
Supplement: Supplementary file 1 [file ijms-20-04176-s001.pdf]

International Journal of
Molecular Sciences

MDPI

Intragenic mutations of FOXG1-related encephalopathy reported in the literature.

Genetic mutations (amino acid change)	*FOXG1* domain	Coding effect	References
c.133_469del337insACCCACCGCCCC ACCCACCGCCCC (p.Pro45Thrfs*39)	N-terminal	Frameshift	[1,2]
c.136C>T (p.Gln46Ter)	N-terminal	Nonsense	[3]
c.201_224del24 (p.Pro66_Gln73del)	N-terminal	Frameshift	[4]
c.214C>T (p.Gln72Ter)	N-terminal	Nonsense	[5]
p.Gln73dup	N-terminal	In-frame	[6]
c.222_223dupGC (p.Pro75Argfs*118)	N-terminal	Frameshift	[1]
c.256dupC (p.Gln86Profs*34)	N-terminal	Frameshift	[7,8]
p.Gln86Profs*35	N-terminal	Frameshift	[6]
c.256delC (p.Gln86Argfs*106)	N-terminal	Frameshift	[8,9]
c.256C>T (p.Gln86Ter)	N-terminal	Nonsense	[1,5,10]
c.263_278del16 (p.Arg88Profs*99)	N-terminal	Frameshift	[10,11]
c.292_295delGGCC (p.Gly98Profs*93)	N-terminal	Frameshift	[4]
c.298del (p.Gln100Serfs*92)	N-terminal	Frameshift	[12]
c.385delG (p. Glu129Serfs*63)	N-terminal	Frameshift	[5]
c.392delG (p.Gly131Alafs*60)	N-terminal	Frameshift	[13]
c.406G>T (p.Glu136Ter)	N-terminal	Nonsense	[5]
p.Glu136Glyfs*39	N-terminal	Frameshift	[6]
c.430G>T (p.Glu144Ter)	N-terminal	Nonsense	[1]
c.460G>T (p.Glu154Ter)	N-terminal	Nonsense	[14]
c.460delG (p.Glu154Argfs*38)	N-terminal	Frameshift	[5]
c.460dupG (p.Glu154Glyfs*301)	N-terminal	Frameshift	[5,10,11,15]
c.461insG (p.Glu155Glyfs*300)	N-terminal	Frameshift	[4,6]
p.Glu155Ter	N-terminal	Nonsense	[6]
p.Lys162Serfs*51	N-terminal	Frameshift	[6]
c.505_506delGGinsT(p.Gly168Serfs*23)	N-terminal	Frameshift	[10]
c.506delG (p.Gly169Alafs*23)	N-terminal	Frameshift	[1]
c.506dup (p.Lys170Glnfs*285)	N-terminal	Frameshift	[16]

c.515_577del63 (p.Gly172_Met192del)	N-terminal	In-frame	[1,15,17]
c.517G>T (p.Glu173Ter)	N-terminal	Nonsense	[5]
c.537C>G (p.Tyr179Ter)	N-terminal	Nonsense	[6]
c.541A>G (p.Lys181Glu)	forkhead cs	Missense	[4]
c.543G>C (p.Lys181Asn)	forkhead cs	Missense	[5]
p.Pro182Leu	forkhead cs	Missense	[6]
c.545C>A (p.Pro182Gln)	forkhead cs	Missense	[5]
c.552dupC (p.Ser185Glnfs*270)	forkhead cs	Frameshift	[6,18]
c.553A>T (p.Ser185Cys)	forkhead cs	Missense	[5]
c.559A>G (p.Asn187Asp)	forkhead cs	Missense	[6,19]
c.561C>A (p.Asn187Lys)	forkhead cs	Missense	[5,6]
c.565C>T (p.Leu189Phe)	forkhead cs	Missense	[5]
c.565C>G (p.Leu189Val)	forkhead cs	Missense	[20]
c.569T>A (p. Ile190Asn)	forkhead cs	Missense	[21]
c.572T>G (p.Met191Arg)	forkhead cs	Missense	[11,15]
c.577G>A (p.Ala193Thr)	forkhead cs	Missense	[1,22]
p.Ile194Serfs*19	forkhead cs	Frameshift	[6]
c.581T>G (p.Ile194Ser)	forkhead cs	Missense	[5]
p.Arg195Pro	forkhead	Missense	[6]
c.586C>T (p.Gln196Ter)	forkhead	Nonsense	[1,6]
c.590G>T (p.Ser197Ile)	forkhead	Missense	[5]
c.592_594delCCC (p.Prp198del)	forkhead	In-frame	[5]
c.609_616del (p.L204Hfs*248)	forkhead	Frameshift	[14]
c.610C>T (p.Leu204Phe)	forkhead	Missense	[6,23]
c.619A>T (p.Ile207Phe)	Forkhead	Missense	[4]
c.624C>G (p.Tyr208Ter)	forkhead	Nonsense	[4,5,18]
p.Tyr208_Ile211del	forkhead	In-frame	[6]
c.643T>C (p.Phe215Leu)	forkhead	Nonsense	[18]
c.651C>G (p.Tyr217Ter)	forkhead	Nonsense	[1]
c.653A>C (p.Tyr218Ser)	forkhead	Missense	[4]
p.Gly224Ser	forkhead	Missense	[6]

c.681C > G (p.Asn227Lys)	forkhead	Missense	[18]
c.688C>G (p.Arg230Gly)	forkhead	Missense	[4]
c.689G>A (p.Arg230His)	forkhead	Missense	[6,24]
c.693C>A (p.Hys231Gln)	forkhead	Missense	[4]
c.694A>T (p.Asn232Tyr)	forkhead	Missense	[25]
c.695A>G (p.Asn232Ser)	forkhead	Missense	[11]
c.700T>C (p.Ser234Pro)	forkhead	Missense	[10]
p.Val242Cysfs*84	forkhead	Frameshift	[6]
c.730C>T (p.Arg244Cys)	forkhead	Missense	[26]
c.732_741del10 (p.His245Thrfs*78)	forkhead	Frameshift	[5]
c.735delC (p.Tyr246Thrfs*80)	forkhead	Frameshift	[1]
c.755G>T (p.Gly252Val)	forkhead	Missense	[1,6]
c.755G>A (p.Gly252Asp)	forkhead	Missense	[5]
c.757A>G (p.Asn253Asp)	forkhead	Missense	[10]
c.762C>G (p.Tyr254Ter)	forkhead	Nonsense	[1]
p.Tyr254Thrfs*72	forkhead	Frameshift	[6]
c.763T>C (p.Trp255Arg)	forkhead	Missense	[6,8]
c.765G>A (p.Trp255Ter)	forkhead	Missense	[27]
p.Leu257Pro	forkhead	Missense	[6]
c.775C>T (p.Pro259Ser)	forkhead	Missense	[25]
c.788_792delACGTG(p.Asp263Valfs*190)	forkhead	Frameshift	[28]
p.Ile266Tyrfs*189	forkhead	Frameshift	[6]
c.858dupC (p.Lys287Glnfs*168)	C-terminal	Frameshift	[29]
c.893C>A (p.Thr298Asn)	C-terminal	Missense	[25]
c.921C>G (p.Try307Ter)	C-terminal	Nonsense	[5]
c.924G>A (p.Trp308Ter)	GBD	Nonsense	[2]
c.946delC (p.Leu316Cysfs*10)	GBD	Frameshift	[11]
c.953_954insC (p.Arg320Profs*135)	C-terminal	Frameshift	[4]
c.969delC (p.Ser323Argfs*3)	C-terminal	Frameshift	[27]
c.974dupT (p.Leu325Phefs*130)	C-terminal	Frameshift	[5]
c.974_975insA (p.Ser326Glufs*129)	C-terminal	Frameshift	[6,30]

c.981C>A (p.Tyr327Ter)	C-terminal	Nonsense	[11]
c.1082dup (p.Leu362Profs*93)	C-terminal	Frameshift	[5]
c.1117_1118insCGTA(p.Ala375Valfs*81)	C-terminal	Frameshift	[4]
c.1141delG (p.Ala381Profs*4)	C-terminal	Frameshift	[5]
c.1186C>A, (p.Cys396Ter)	JBD	Nonsense	[11]
c.1200C>A (p.Tyr400Ter)	JBD	Nonsense	[3]
c.1200C>G (p.Tyr400Ter)	JBD	Nonsense	[2]
p.Asn408Ile	C-terminal	Missense	[6]
c.1248C>G (p.Tyr416Ter)	C-terminal	Missense	[14]
c.1470A>C (p.Ter490Tyrext*21)	C-terminal	Stop loss	[4]

[1] Table S1 shows the published ***FOXG1*** variants.

References

1. Seltzer, L.E.; Ma, M.; Ahmed, S.; Bertrand, M.; Dobyns, W.B.; Wheless, J.; Paciorkowski, A.R. Epilepsy and outcome in FOXG1-related disorders. *Epilepsia* **2014**, *55*, 1292–1300, doi:10.1111/epi.12648.
2. Philippe, C.; Amsallem, D.; Francannet, C.; Lambert, L.; Saunier, A.; Verneau, F.; Jonveaux, P. Phenotypic variability in Rett syndrome associated with FOXG1 mutations in females. *J. Med. Genet.* **2010**, *47*, 59–65, doi:10.1136/jmg.2009.067355.
3. De Filippis, R.; Pancrazi, L.; Bjørgo, K.; Rosseto, A.; Kleefstra, T.; Grillo, E.; Panighini, A.; Cardarelli, F.; Meloni, I.; Ariani, F., et al. *Expanding the phenotype associated with FOXG1 mutations and in vivo FoxG1 chromatin-binding dynamics*; 2011; Vol. 82, pp. 395-403.
4. Vidal, S.; Brandi, N.; Pacheco, P.; Gerotina, E.; Blasco, L.; Trotta, J.R.; Derdak, S.; Del Mar O'Callaghan, M.; Garcia-Cazorla, A.; Pineda, M., et al. The utility of Next Generation Sequencing for molecular diagnostics in Rett syndrome. *Sci. Rep.* **2017**, *7*, 12288, doi:10.1038/s41598-017-11620-3.
5. Mitter, D.; Pringsheim, M.; Kaulisch, M.; Plumacher, K.S.; Schroder, S.; Warthemann, R.; Abou Jamra, R.; Baethmann, M.; Bast, T.; Buttel, H.M., et al. FOXG1 syndrome: genotype-phenotype association in 83 patients with FOXG1 variants. *Genet. Med.* **2017**, 10.1038/gim.2017.75, doi:10.1038/gim.2017.75.
6. Vegas, N.; Cavallin, M.; Maillard, C.; Boddaert, N.; Toulouse, J.; Schaefer, E.; Lerman-Sagie, T.; Lev, D.; Magalie, B.; Moutton, S., et al. Delineating FOXG1 syndrome: From congenital microcephaly to hyperkinetic encephalopathy. *Neurology. Genet.* **2018**, *4*, e281, doi:10.1212/nxg.0000000000000281.
7. Le Guen, T.; Bahi-Buisson, N.; Nectoux, J.; Boddaert, N.; Fichou, Y.; Diebold, B.; Desguerre, I.; Raqbi, F.; Daire, V.C.; Chelly, J., et al. A FOXG1 mutation in a boy with congenital variant of Rett syndrome. *Neurogenetics* **2011**, *12*, 1–8, doi:10.1007/s10048-010-0255-4.
8. Wong, L.-C.; Wu, Y.-T.; Hsu, C.-J.; Weng, W.-C.; Tsai, W.-C.; Lee, W.-T. Cognition and Evolution of Movement Disorders of FOXG1-Related Syndrome. *Front. Neurol.* **2019**, *10*, doi:10.3389/fneur.2019.00641.
9. Allou, L.; Lambert, L.; Amsallem, D.; Bieth, E.; Edery, P.; Destree, A.; Rivier, F.; Amor, D.; Thompson, E.; Nicholl, J., et al. 14q12 and severe Rett-like phenotypes: new clinical insights and physical mapping of FOXG1-regulatory elements. *Eur. J. Hum. Genet.* **2012**, *20*, 1216–1223, doi:10.1038/ejhg.2012.127.

10. Kortüm, F.; Das, S.; Flindt, M.; Morris-Rosendahl, D.J.; Stefanova, I.; Goldstein, A.; Horn, D.; Klopocki, E.; Kluger, G.; Martin, P. The core FOXG1 syndrome phenotype consists of postnatal microcephaly, severe mental retardation, absent language, dyskinesia, and corpus callosum hypogenesis. *J. Med. Genet.* **2011**, jmg. 2010.087528.
11. Papandreou, A.; Schneider, R.B.; Augustine, E.F.; Ng, J.; Mankad, K.; Meyer, E.; McTague, A.; Ngoh, A.; Hemingway, C.; Robinson, R., et al. Delineation of the movement disorders associated with FOXG1 mutations. *Neurology* **2016**, *86*, 1794–1800, doi:10.1212/wnl.0000000000002585.
12. Helbig, K.L.; Farwell Hagman, K.D.; Shinde, D.N.; Mroske, C.; Powis, Z.; Li, S.; Tang, S.; Helbig, I. Diagnostic exome sequencing provides a molecular diagnosis for a significant proportion of patients with epilepsy. *Genet. Med.* **2016**, *18*, 898–905, doi:10.1038/gim.2015.186.
13. Caporali, C.; Signorini, S.; De Giorgis, V.; Pichiecchio, A.; Zuffardi, O.; Orcesi, S. Early-onset movement disorder as diagnostic marker in genetic syndromes: Three cases of FOXG1-related syndrome. *Eur. J. Paediatr. Neurol.* **2018**, *22*, 336–339, doi:10.1016/j.ejpn.2018.01.007.
14. Bahi-Buisson, N.; Nectoux, J.; Girard, B.; Van Esch, H.; De Ravel, T.; Boddaert, N.; Plouin, P.; Rio, M.; Fichou, Y.; Chelly, J., et al. Revisiting the phenotype associated with FOXG1 mutations: two novel cases of congenital Rett variant. *Neurogenetics* **2010**, *11*, 241–249, doi:10.1007/s10048-009-0220-2.
15. McMahon, K.Q.; Papandreou, A.; Ma, M.; Barry, B.J.; Mirzaa, G.M.; Dobyns, W.B.; Scott, R.H.; Trump, N.; Kurian, M.A.; Paciorkowski, A.R. Familial recurrences of FOXG1-related disorder: Evidence for mosaicism. *Am. J. Med. Genet. A* **2015**, *167a*, 3096–3102, doi:10.1002/ajmg.a.37353.
16. De Bruyn, C.; Vanderhasselt, T.; Tanyalcin, I.; Keymolen, K.; Van Rompaey, K.L.; De Meirleir, L.; Jansen, A.C. Thin genu of the corpus callosum points to mutation in FOXG1 in a child with acquired microcephaly, trigonocephaly, and intellectual developmental disorder: a case report and review of literature. *Eur. J. Paediatr. Neurol.* **2014**, *18*, 420–426, doi:10.1016/j.ejpn.2013.11.010.
17. Ma, M.; Adams, H.R.; Seltzer, L.E.; Dobyns, W.B.; Paciorkowski, A.R. Phenotype Differentiation of FOXG1 and MECP2 Disorders: A New Method for Characterization of Developmental Encephalopathies. *J. Pediatr.* **2016**, *178*, 233–240.e210, doi:10.1016/j.jpeds.2016.08.032.
18. Mencarelli, M.A.; Spanhol-Rosseto, A.; Artuso, R.; Rondinella, D.; De Filippis, R.; Bahi-Buisson, N.; Nectoux, J.; Rubinsztajn, R.; Bienvenu, T.; Moncla, A., et al. Novel FOXG1 mutations associated with the congenital variant of Rett syndrome. *J. Med. Genet.* **2010**, *47*, 49–53, doi:10.1136/jmg.2009.067884.
19. Terrone, G.; Bienvenu, T.; Germanaud, D.; Barthez-Carpentier, M.A.; Diebold, B.; Delanoe, C.; Passemard, S.; Auvin, S. A case of Lennox-Gastaut syndrome in a patient with FOXG1-related disorder. *Epilepsia* **2014**, *55*, e116–e119, doi:10.1111/epi.12800.
20. Olson, H.E.; Tambunan, D.; LaCoursiere, C.; Goldenberg, M.; Pinsky, R.; Martin, E.; Ho, E.; Khwaja, O.; Kaufmann, W.E.; Poduri, A. Mutations in epilepsy and intellectual disability genes in patients with features of Rett syndrome. *Am. J. Med. Genet. A* **2015**, *167a*, 2017–2025, doi:10.1002/ajmg.a.37132.
21. Harada, K.; Yamamoto, M.; Konishi, Y.; Koyano, K.; Takahashi, S.; Namba, M.; Kusaka, T. Hypoplastic hippocampus in atypical Rett syndrome with a novel FOXG1 mutation. *Brain Dev.* **2018**, *40*, 49–52.
22. Van der Aa, N.; Van den Bergh, M.; Ponomarenko, N.; Verstraete, L.; Ceulemans, B.; Storm, K. Analysis of FOXG1 Is Highly Recommended in Male and Female Patients with Rett Syndrome. *Mol Syndr.* **2011**, *1*, 290–293, doi:10.1159/000330755.
23. Meneret, A.; Mignot, C.; An, I.; Habert, M.O.; Jacquette, A.; Vidailhet, M.; Bienvenu, T.; Roze, E. Generalized dystonia, athetosis, and parkinsonism associated with FOXG1 mutation. *Mov. Disord.* **2012**, *27*, 160–161, doi:10.1002/mds.23956.
24. Takahashi, S.; Matsumoto, N.; Okayama, A.; Suzuki, N.; Araki, A.; Okajima, K.; Tanaka, H.; Miyamoto, A. FOXG1 mutations in Japanese patients with the congenital variant of Rett syndrome. *Clin. Genet.* **2012**, *82*, 569–573, doi:10.1111/j.1399-0004.2011.01819.x.
25. Zhang, Q.; Yang, X.; Wang, J.; Li, J.; Wu, Q.; Wen, Y.; Zhao, Y.; Zhang, X.; Yao, H.; Wu, X., et al. Genomic mosaicism in the pathogenesis and inheritance of a Rett syndrome cohort. *Genet. Med.* **2019**, *21*, 1330–1338, doi:10.1038/s41436-018-0348-2.

26. Le Guen, T.; Fichou, Y.; Nectoux, J.; Bahi-Buisson, N.; Rivier, F.; Boddaert, N.; Diebold, B.; Heron, D.; Chelly, J.; Bienvenu, T. A missense mutation within the fork-head domain of the forkhead box G1 Gene (FOXG1) affects its nuclear localization. *Hum. Mutat.* **2011**, *32*, E2026–E2035, doi:10.1002/humu.21422.
27. Ariani, F.; Hayek, G.; Rondinella, D.; Artuso, R.; Mencarelli, M.A.; Spanhol-Rosseto, A.; Pollazzon, M.; Buoni, S.; Spiga, O.; Ricciardi, S., et al. FOXG1 is responsible for the congenital variant of Rett syndrome. *Am. J. Hum. Genet.* **2008**, *83*, 89–93, doi:10.1016/j.ajhg.2008.05.015.
28. Das, D.K.; Jadhav, V.; Ghattargi, V.C.; Udani, V. Novel mutation in forkhead box G1 (FOXG1) gene in an Indian patient with Rett syndrome. *Gene* **2014**, *538*, 109–112, doi:10.1016/j.gene.2013.12.063.
29. Zhang, Q.; Wang, J.; Li, J.; Bao, X.; Zhao, Y.; Zhang, X.; Wei, L.; Wu, X. Novel FOXG1 mutations in Chinese patients with Rett syndrome or Rett-like mental retardation. *Bmc Med. Genet.* **2017**, *18*, 96, doi:10.1186/s12881-017-0455-y.
30. Diebold, B.; Delepine, C.; Nectoux, J.; Bahi-Buisson, N.; Parent, P.; Bienvenu, T. Somatic mosaicism for a FOXG1 mutation: diagnostic implication. *Clin. Genet.* **2014**, *85*, 589–591, doi:10.1111/cge.12212.
